# Binocular Rivalry Impact on Macroblock-Loss Error Concealment for Stereoscopic 3D Video Transmission

**DOI:** 10.3390/s23073604

**Published:** 2023-03-30

**Authors:** Md Mehedi Hasan, Md. Azam Hossain, Naif Alotaibi, John F. Arnold, AKM Azad

**Affiliations:** 1 Department of Robotics and Mechatronics Engineering, University of Dhaka, Dhaka 1000, Bangladesh; mmhasan@du.ac.bd; 2 Department of Computer Science and Engineering, Islamic University of Technology, Gazipur 1704, Bangladesh; 3 Department of Mathematics and Statistics, College of Science, Imam Mohammad Ibn Saud Islamic University (IMSIU), Riyadh 11432, Saudi Arabia; 4 School of Engineering and Information Technology, University of New South Wales, Canberra 2600, Australia

**Keywords:** psychovisual impact, stereoscopic video, statistical analysis, human visual system, macroblock loss

## Abstract

Three-dimensional video services delivered through wireless communication channels have to deal with numerous challenges due to the limitations of both the transmission channel’s bandwidth and receiving devices. Adverse channel conditions, delays, or jitters can result in bit errors and packet losses, which can alter the appearance of stereoscopic 3D (S3D) video. Due to the perception of dissimilar patterns by the two human eyes, they can not be fused into a stable composite pattern in the brain and hence try to dominate by suppressing each other. Thus, a psychovisual sensation that is called binocular rivalry occurs. As a result, undetectable changes causing irritating flickering effects are seen, leading to visual discomforts such as eye strain, headache, nausea, and weariness. This study addresses the observer’s quality of experience (QoE) by analyzing the binocular rivalry impact on the macroblock (MB) losses in a frame and its error propagation due to predictive frame encoding in stereoscopic video transmission systems. To simulate the processing of experimental videos, the Joint Test Model (JM) reference software has been used as it is recommended by the International Telecommunication Union (ITU). Existing error concealing techniques were then applied to the contiguous lost MBs for a variety of transmission impairments. In order to validate the authenticity of the simulated packet loss environment, several objective evaluations were carried out. Standard numbers of subjects were then engaged in the subjective testing of common 3D video sequences. The results were then statistically examined using a standard Student’s t-test, allowing the impact of binocular rivalry to be compared to that of a non-rivalry error condition. The major goal is to assure error-free video communication by minimizing the negative impacts of binocular rivalry and boosting the ability to efficiently integrate 3D video material to improve viewers’ overall QoE.

## 1. Introduction

Due to its immense demand in commercial applications, 3D video transmission has drawn a growing amount of research interest in recent years. A greater emphasis has been made on evaluating and minimizing the consequences of methodologies for 3D image or video capture, processing, rendering, and display in an effort to enhance quality of experience (QoE) [1,2]. Nonetheless, the impacts of artifacts introduced into a 3D video by its transmission method have received less attention than those in a 2D video [3], despite the fact that they affect the overall visual quality in a comparable manner. Errors in transmission over unreliable communication channels are worse for 3D video than for 2D video because 3D video has two separate channels, each of which may suffer uncorrelated impairments; for instance, a delay in one view can result in temporal desynchronization, which can reduce the comfort of 3D viewing [4,5]. However, the techniques used to eliminate these abnormalities (such as error concealment) do not function as well for 3D videos as they do for 2D films [6]. To provide accurate 3D depth perception, the two 3D channels should be maintained and coordinated [7,8] and avoid binocular rivalry [6]. Binocular rivalry refers to the spontaneous, unpredictable changes in perceived awareness induced by different stimulation of the two eyes. The term “Binocular Rivalry” refers to the unpredictable and inconsistent shifts in perceptual awareness that may be triggered by a variety of stimuli presented to each of the two eyes [9,10].

## 2. Background

For a given set of stereoscopic pictures, the weighted average of a 2D and a depth prominent map was calculated to produce a 3D prominent map. These reinforcements from the left and right pictures were selected and fed into a 3D convolutional neural network to assess the discerning quality when the value of 3D prominent map reinforcements is higher than the threshold previously established. The quality scores of the distorted stereoscopic picture were then calculated using a weighted average of the conspicuous image reinforcements. Hu et al. [11] suggested a deep network based on binocular perception to answer the situation of stereoscopic picture quality evaluation, employing four channels, including left view, right view, binocular addition view, and binocular difference view, taking them into account as the input of the network. Feng et al. [12] presented a multiscale-attribute escorted 3D convolutional neural network for assessing stereoscopic video quality. Using the stereoscopic films, they extracted temporal characteristics and gathered multiscale data. They said that their approach demonstrated a strong link with perceptibly detectable human behavior. Stereo perceptible masking under insensible binocular competition was investigated by Zheng et al. [13]. In order to quantify the binocular noise amplitude in different backdrop lustrousness and noise acclimatization pairings, they conducted a psychophysical experiment using the staircase approach. In contrast to ab entirely compatible inclination, they discovered that irreconcilable binocular noise was seen at a greater masking threshold.

The evaluation of quality of service (QoS) and quality of experience (QoE) has been the topic of a significant amount of research, with a particular emphasis placed on video compression standards and communication channel noise. Taha et al. [14] analyzes a number of different network factors that are known to have an influence on quality of experience (QoE), such as packet delay, packet delay variation, and packet loss. The authors have suggested a solution for managing quality of experience (QoE), which is based on machine learning. This system forecasts quality of experience (QoE) requirements and then maintains end-user video quality by delivering optimal services in accordance with the prediction. An adaptive model was presented in the research [2] in order to establish a link between the quantization parameter (QP) of the H.264 and H.265 codecs and the quality of service (QoS) of 5G wireless technology. Nevertheless, because of the limitations, the model that is presented in [2] is unable to allow the development of subsequent generations of codecs. In addition, the consumption of resources (CPU and memory) increases as the complexity of the network topology rises.

In this study, the goal was to engrave the viewers’ quality of experience (QoE) by performing objective evaluations to validate an appropriate simulation packet loss environment and a comprehensive subjective study to elongate the impact of psychovisual binocular rivalry. This was accomplished by analyzing the macroblock (MB) losses in a frame and its error propagation caused by predictive frame encoding while stereoscopic videos were being transmitted.

### 2.1. 3D Visual Perception

The impression of depth in stereoscopic 3D video is dependent on how the human brain and eyes function. Two pictures representing two views of the same object appear independently to the eyes, with human vision interpreting the difference (disparity) between them to generate a depth feeling in the brain, which experiences a single image known as the cyclopean view [15]. As a consequence of the eyes’ horizontal separation, disparity refers to the difference in the picture positions of an item perceived by the left and right eyes. In stereopsis, the brain extracts depth information from two-dimensional (2D) retinal pictures via binocular disparity. Binocular disparity in computer vision is the difference between the coordinates of comparable features in two stereo pictures.

**Figure 1:**
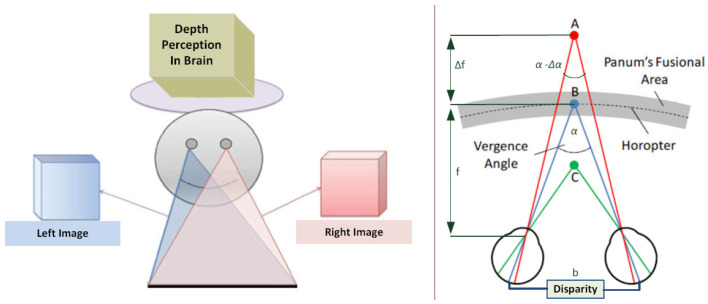
Disparity in a binocular vision and the geometry of occured depth perception in our brain [9].

As illustrated in Figure 1, a curving line that links all locations with zero retinal disparity (same relative coordinate) is referred to as the horopter, and the points positioned at it have the same perceived distance from a human subject’s fixation point. Panum’s fusional area is a zone around the horopter in which objects with non-zero retinal disparities may be merged binocularly, but items positioned outside of this region produce double pictures. Howard et al. [16] demonstrates that the size of Panum’s area is not constant throughout the retina and is dependent on the spatial and temporal features of the fixation object. As a person focuses on an item, the picture of that object falls on the retina, and things closer to or farther from the accommodation distance should seem hazy. While the human visual system (HVS) is tolerant of a tiny degree of blur, objects that lie within a limited zone surrounding the accommodation point may be regarded as having high resolution (i.e., not blurred), with the size of this region known as the depth of field (DOF) [16].

At a fixation point of *B*, as shown in Figure 1, the stereopsis geometry matches the experimental setting of the two-needle test [17]. It states that the theoretical depth discrimination Δf may be calculated using the convergence angle α by dividing by the depth difference df (dα/df), which provides

(1) $$\Delta f=-\frac{f^{2}}{b}\left(1+\frac{b^{2}}{{4f}^{2}}\right)\cdot \Delta \alpha$$

with *b* representing the inter-pupillary distance and *f* representing the average object distance. With $4f^{2}\gg b^{2}$, which is true even for a close point distance of $f_{near}$ = 250 mm, Equation (1) reduces to the standard form $\Delta f\approx -f^{2}\frac{\Delta \alpha }{b}$, which is the stereoscopic acuity (the lowest observable depth difference) necessary for binocular or 3D depth perception in binocular vision. It is observed scientifically that Δα=10arcsec (a value acceptable under photopic lighting circumstances), which is added to transfer the vision from fixation point A (α−Δα) to (α) and for greater depth perception B (α) to C (α+Δα). The least discernible depth difference at a fixation distance of 650 mm is on the order of 0.3 mm. In addition to other aspects, stereoscopic acuity is primarily impacted by an object’s brightness and spatial frequency, as well as angular distances from fixation and object motion. Therefore, it is necessary to build a multidimensional 3D visual experience model that incorporates the aforementioned acuity parameters based on their perceptual significance.

### 2.2. Binocular Rivalry

**Figure 2:**
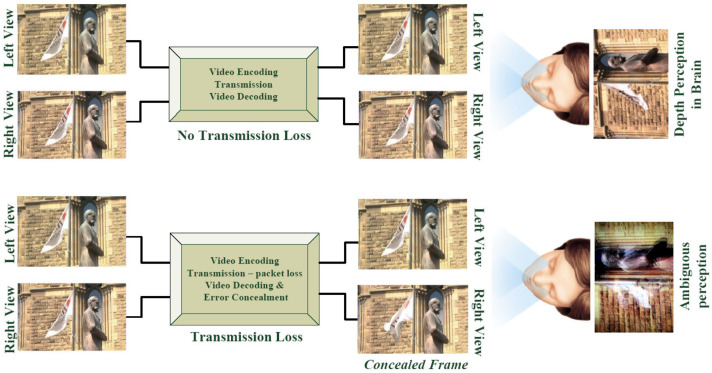
Binocular rivalry: perceptual ambiguity caused by transmission loss.

The only form of binocular interaction that can be described by the suppression theory of binocular vision is the rivalry [18] type, which operates for both similar and distinct images. It is a visual phenomenon that occurs, as seen in Figure 2, when separate monocular stimuli are delivered to corresponding retinal areas on both eyes. In accordance with the binocular suppression hypothesis [19], the better view (i.e., the left or right) typically determines the overall perception, provided that the quality of the poorer view exceeds a threshold value. Nevertheless, this potential is limited, since research has shown that an additional cognitive load [18] is necessary to merge various views, causing eye strain and visual fatigue and preventing individuals from seeing 3D information for lengthy periods of time. This issue resulted in the shutdown of various 3D TV channels, including *ESPN 3D*, *Foxtel 3D*, *N3D*, and *3NET* [20], and the limitation of broadcasting services, including *MSG 3D* and *Sky 3D* channels.

### 2.3. Binocular Rivalry in S3D Transmission

The impact and modeling of binocular rivalry in stereoscopic videos is in an emerging stage, and there are two issues that need to be clarified: (1) the types of inter-view picture changes that might generate binocular rivalry and (2) how it impacts both 3D visual comfort and video quality. It is often difficult to capture binocular rivalry artifacts in a stereoscopic pair due to the fact that the pictures received by both eyes are merged into a single 3D image. In addition, it is difficult to exactly characterize or depict the signals employed by the HVS to generate depth perception in the human brain, as well as their associated properties. A good 3D depth perception involves the probabilistic integration of a number of depth signals, such as binocular disparity, and binocular and monocular 3D cues, such as accommodation, convergence, parallax, occlusion between objects, and perspective relative size. In addition, these cues and their interpretations must be kept in 3D material throughout transmission and at the display decoder; otherwise, a collision of binocular cues would quickly cause inconsistencies and annoyances. Recent research has thus focused on determining the relative strength of these signals and comparing them to stereoscopic disparity. The following characteristics impede 3D depth perception and generate binocular ambiguities, which are the primary causes of visual discomfort and video quality degradation:

- In any perspective of a 3D video frame, video coding and compression artifacts (such as blocking, staircase, ringing, mosaic, motion, and blurring) may occur and cause distortions. Modern picture processing and encoding methods, however, have lessened these issues.
- In transmission network channels, queuing, congestion, and serialization effects generate variations in packet transit delays known as jitter. Higher degrees of jitter may cause binocular ambiguity between views because the packets for either view are received too late for their needed display time. In general, larger levels of jitter are more likely to occur in either sluggish or highly crowded channels during transmission.
- A packet loss in the network might cause the decoder to fail to give accurate left and right pictures, which can lead to data loss and binocular mismatch. This discrepancy may cause spatial aliasing, color bleeding, motion artifacts, intensity discrepancies, and other problems [21].
- The decoder synchronization necessary to recover losses and the packet delay brought on by jitter in either of the two views can produce binocular rivalry artifacts that impair accurate depth perception.
- The implementation of existing 2D error concealment approaches for 3D distorted videos also introduces binocular artifacts and can result in binocular rivalry.
- At the acquisition and display phases of a 3D transmission chain, stereoscopic distortions may cause binocular rivalry. Quick variations in object depth caused by motion might result in fast vergence shifts that viewers must track. Additionally, an observer may have crisp and double vision of ordinarily indistinct objects, while abrupt scene changes affect the observer’s impression of the depth of the scene, requiring a re-adaptation of the observer’s vision.
- A cardboard effect stereoscopic distortion provides an artificial depth impression in which things look flat, as if the scene had been separated into distinct depth planes [17]. In addition, the absence of delicate visual elements creates a puppet-theater effect [22].

In conclusion, binocular vision is very susceptible to ambiguous stereo pairings being presented to the human eye and creating binocular rivalry artifacts, which may cause significant visual discomfort and lead to headache, eye strain, nausea, simulator sickness [23], and visual fatigue.

### 2.4. Macroblock Loss in S3D Transmission

**Figure 3:**
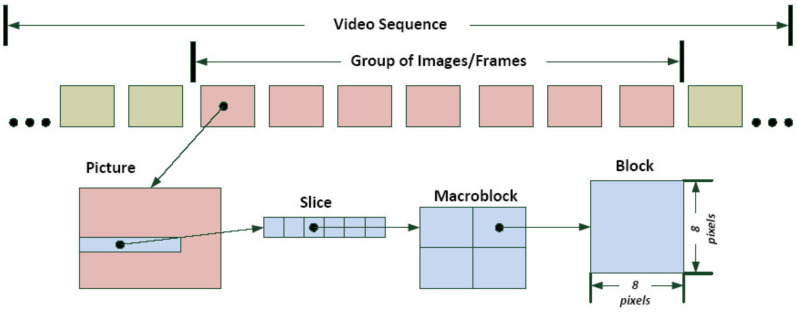
Partitioning of macroblocks (MBs) for inter-frame prediction.

Similar to network impairments in 2D video transmission, 3D channels suffer from one or more frame losses and MB or slice losses [24], which have negative effects on the quality of the received service. Figure 3 depicts the MB and block structures used as the foundation for motion-compensated prediction in H.264 video coding to decrease the temporal correlation between frames. If there is a network delay, jitter, or packet loss, one or more megabytes of data are lost. Figure 3 demonstrates that the frames or images in a video are composed of slices, each of which is a group of rows of MBs that are composed of blocks of pixels. These hierarchical structures make it easy to arrange compressed video content packaging.

The kind of stereoscopic picture quality loss brought on by lost macroblocks (MBs) or slices is not equivalent to that brought on by quantization or low-pass filtering, which was researched in earlier publications [25,26]. The loss of a block or MB of data has a major influence on the binocular perception of a stereo picture pair. Standard error concealing algorithms are not suitable for stereoscopic pictures [27] because a block must be matched (based on disparity) with the block from the opposite view to provide a correct 3D impression. Even moderately effective monoscopic concealments may cause severe stereoscopic visual distortions. The chapter [28] provides a short description of recently developed 3D error hiding techniques. Our most recent research and experiment results [29], demonstrate that, in addition to transmission losses, using traditional 2D error concealment methods in a 3D video decoder also causes these artifacts and poses a perceptual challenge, necessitating an analysis to ascertain the psychovisual effects. We concentrate on creating network losses and using current error concealment technologies to evaluate their overall impact since the goal of this research was to ascertain the psychovisual impact of error concealment following the loss of one or more MBs. We try to broaden this by using the Student’s *t*-test [30] to analyze and compare for various error propagation scenarios in order to define the statistical significance of binocular rivalry in the former when error concealment is applied independently to each view. This is because most researchers have limited their subjective analysis to taking into account the mean opinion score (MOS) and confidence interval (CI).

## 3. Materials and Methods

**Figure 4:**
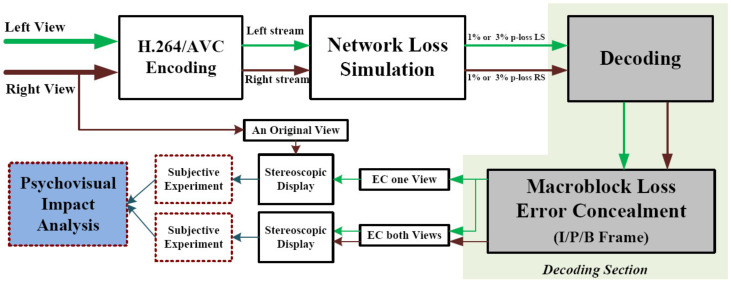
Block diagram of the proposed model.

In order to investigate the total psychovisual effect of MB losses in one view and identical portions of both, simulations of various MB and slice losses conducted in the JM reference platform and conventional error concealing procedures are applied and addressed in this part as shown in Figure 4. The approach is explored in more depth in subsequent subsections.

### 3.1. Proposed Video Processing Chain

**Figure 5:**
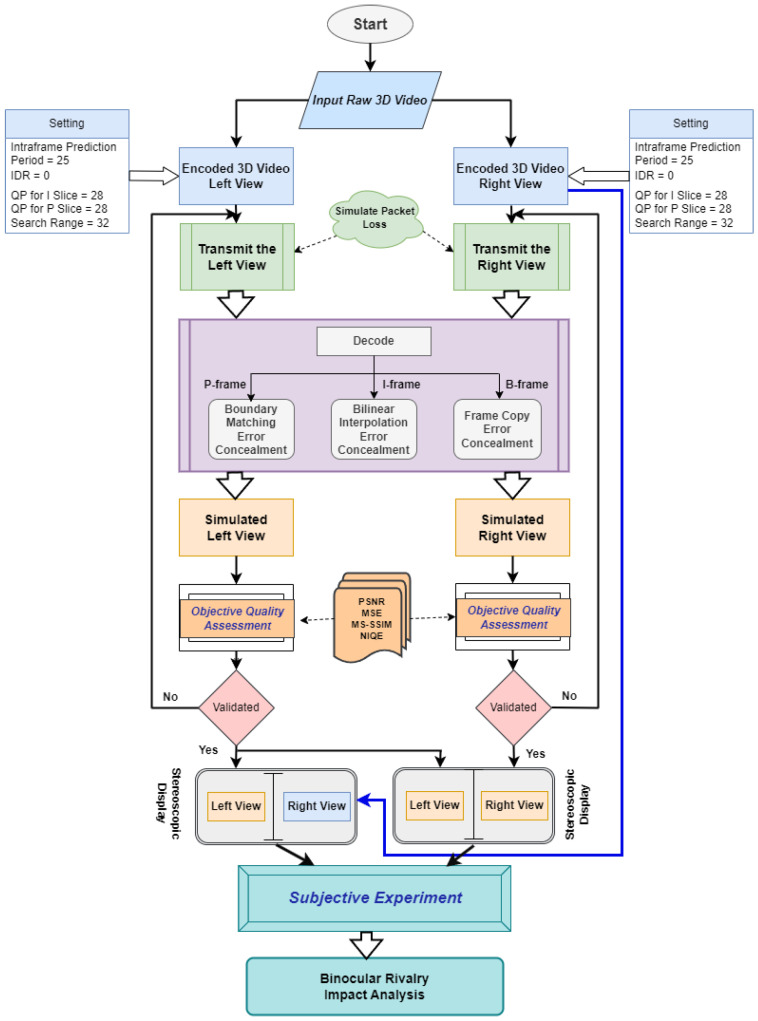
Block diagram of the proposed 3D video processing chain and evaluation of transmitted error-concealed videos.

The detailed flow-graph of the overall processing chain used to encode the stereo video, generate transmission losses, and apply error concealment techniques at the decoder end is shown in Figure 5. These sequences were encoded in accordance with the guidelines used in the H.264 JM 18.6 [31] reference platform. Several different scenarios, referred to as hypothetical reference circuits (HRCs) in accordance with the terminology of the Video Quality Experts Group (VQEG) [32], were used to create them. Round transport protocol (RTP) packets and conventional MB-loss error hiding methods are used at the decoder end to mimic data losses in the network.

After that, a group of individuals is asked to evaluate the transmitted stereoscopic videos’ video quality and visual comfort levels under certain system circumstances in order to determine the overall effects. The analysis of the transmission process as a whole, including the 3D video display, subjective assessment, and psychovisual effect, is conducted in the evaluation portion.

### 3.2. Video Coding and Transmission

The ITU and ISO/IEC MPEG’s cutting-edge video coding standard, H.264/AVC, aims to increase coding effectiveness and improve network adaptability. A video coding layer (VCL) and a network abstraction layer (NAL) make up its two conceptual levels . Although the latter establishes the interface between the encoded video data and transport medium, the former deals with the effective encoding of the video data. H.264/AVC may partition an image into slices in order to strengthen the resilience of the bit stream against transmission defects at the expense of higher overhead. Erroneous slices are rejected before being sent to the decoder, and the incoming data are delayed so that they may be retrieved in the proper decoding sequence. According to Figure 3, the slice header provides details about the image to which it belongs, the locations of the encoded MBs, and the hierarchical structure. A image may be split up into many slices by an encoder, increasing the bit stream’s resistance against transmission defects. Each slice carries its own local information to allow independent decoding. Slice losses may be handled by the simulation environment; the topic of their concealment will be covered later. All algorithms, however, often deal with instances when just a little amount of a received video frame is absent, such as a single or a series of consecutive MBs. The details of the commands and configurations of the encoding and decoding processes are included in Appendix A.

### 3.3. Frame Predictive Error Concealment

**Figure 6:**
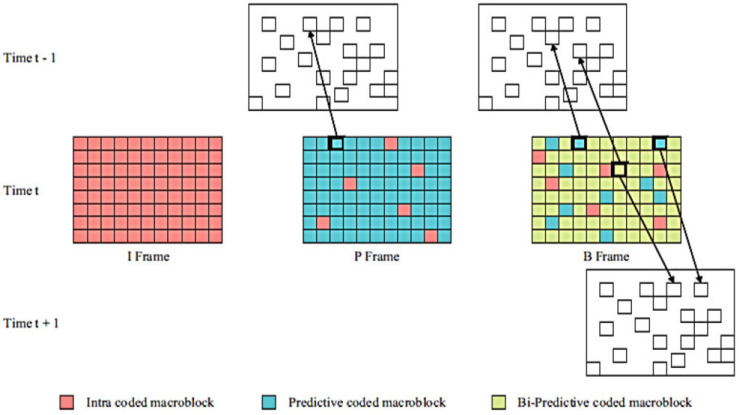
Different frame predictions in H.264/AVC encoding.

The H.264/AVC standard includes the non-normative error concealing function, which effectively increases fault resilience at the decoder without adding any more data to the bit stream. The majority of transmission faults should be detectable and hidden by an error-resilient decoder. The error concealment techniques in H.264/AVC may typically be classified into two groups of spatial and temporal concealments depending on the information utilised. In order to recover the error areas in a frame, spatial error concealment leverages spatial redundancy. Figure 6 shows how alternative inter-view frame predictions may be used by H.264/AVC coding to encode a video frame. Temporal adjacent frames are used in temporal error concealing to hide flaws in the current frame. In our research, the following error concealing techniques were used for various types of prediction frames.

#### 3.3.1. Bilinear Interpolation for I-frame

The easiest technique to hide any parts of an I-frame damaged by packet loss is to use a traditional spatial interpolation algorithm because there is no inter-frame prediction in an I-frame [33]. In order to help with bilinear interpolation, Sobel edge detection [34] is based on the construction of a reliable Hough-transform-based approach [35] that can systematically join edges, regardless of the number of edge points around empty regions, using the following steps:

- Step 1: Choose n adjacent neighbors around the missing packet (e.g., n lines above and below the lost area).
- Step 2: Identify all the cells whose peaks exceed a specified threshold (tp). (The presence of a straight line in the picture is indicated by a peak in the accumulator array. The Hough transform’s ability to connect edges relies on the size of the accumulator cell used to identify the greatest peaks.)
- Step 3: Combine adjacent cells with similar Hough parameters into a single cell.
- Step 4: In order to join the jagged edges, use the inverse Hough transform.

**Figure 7:**
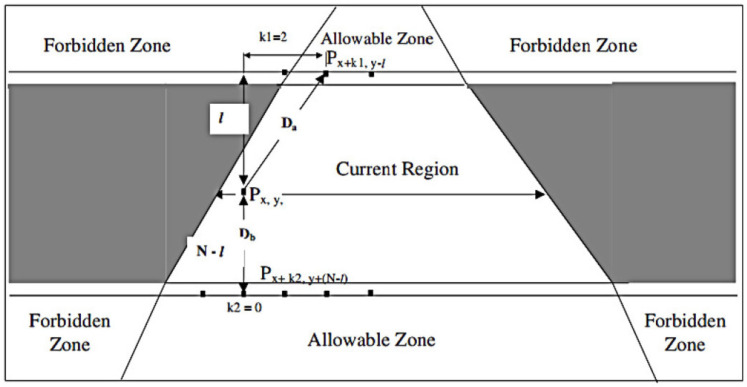
Region-based interpolation used for error concealment in I-frame.

The missing portions are divided into several regions for interpolation along the direction of each identified line using the connecting edges. The missing portions must then be divided into several sections to facilitate interpolation, as shown in Figure 7. The nearest reference pixels in the same zone [33] were subjected to bilinear interpolation by Gharavi et al., who utilized their weights for interpolation as

(2) $$P_{x,y}=\frac{D_{b}}{D_{a}+D_{b}}P_{x+k1,y-l}+\frac{D_{a}}{D_{a}+D_{b}}P_{x+k2,y+(N-l)}$$

where $D_{a}$ and $D_{b}$ are the distances between the interpolating pixel $P_{x,y}$ and its same-zone bilinear reference pixels $P_{x+k1,y-l}$ and $P_{x+k2,y+(N-l)}$, respectively. The latter are the nearest pixels to $P_{x,y}$ located above and below the missing area at the horizontal distances of k1 and k2 and vertical ones of *l* and (N−l), respectively, where *N* is the height of the missing area (e.g., MB).

#### 3.3.2. Boundary Matching for P-Frame

**Figure 8:**
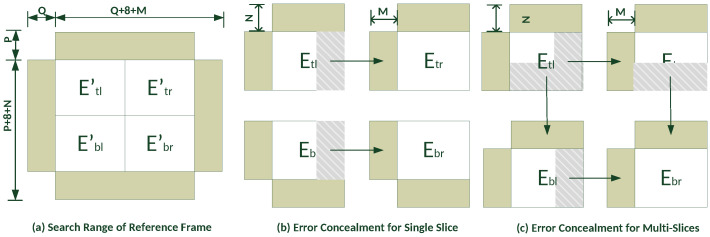
Boundary-matching algorithm for error concealment in P-frame.

For error hiding in P-frames, the spatio-temporal boundary-matching method [36] is used. Temporal information may be utilized to hide lost slices or missing MBs since a P-frame can be anticipated from either an I- or P-frame. Su et al., (2006) used the multiside boundary-matching approach for error hiding. The suggested approach breaks a damaged MB into four 8 × 8 sub-blocks and utilizes the information in the adjacent rows and columns to disguise the MB, as illustrated in Figure 8.

The nearest adjacent pixels in the N rows and M columns of the MB are used to find the best block for recovering the MB. In Figure 8, $E_{tl}$, $E_{tr}$,$E_{bl}$, and $E_{br}$ are the blocks divided at different locations. To conceal a block, N rows and M columns from its top/bottom and left/right boundaries, respectively, were used. The search range (P/Q) was set to 32 and a full search (FS) was used to evaluate all possible candidate sub-blocks within it, as shown in Figure 8a. Then, the best block for recovering a lost one was determined by the minimum sum of the absolute difference (SAD), expressed as

(3) $$\begin{array}{cc} SAD(E_{tl}) & =\sum_{j=-N}^{-1}\sum_{i=0}^{7}|E_{tl}\left(x+i,y+j\right)-E_{tl}^{{}^{\prime }}\left(x+dx+i,y+dy+j\right)|\\ \; & +\sum_{j=0}^{7}\sum_{i=-M}^{-1}|E_{tl}\left(x+i,y+j\right)-E_{tl}^{{}^{\prime }}\left(x+dx+i,y+dy+j\right)| \end{array}$$

where *x* and *y* are the top- and left-most positions, respectively, of the lost sub-block in the current frame, and dx and dy are the displacements between that sub-block and the candidate one in the reference frame. Sub-block $E_{tl}$ is recovered by the block with the minimum SAD (Equation (3)) and sub-blocks $E_{tr}$,$E_{bl}$, and $E_{br}$ concealed using the same strategy. When concealing these kinds of errors, as the neighboring blocks of a corrupted block may also be lost, the different error concealment methods for single and multiple slices proposed in [37] are shown in Figure 8b,c, respectively.

#### 3.3.3. Frame Copy for B-Frame

For the purpose of reconstructing the pixels lost in B-frames, frame-copy error concealment is used. In our approach, I-/P-frames are used to forecast B-frames via hierarchical coding. As a B-frame cannot foresee the next frame, using pixels from the same positions in the previous frame is a frequent technique for hiding missing pixels. In order to recover quickly during real-time transmission, a decoder may sometimes throw away a whole frame and replace it with the one before it. Instead of designing a better error concealment method, our major objective was to study the psychovisual effects of 2D error concealment techniques used in stereoscopic recordings that were hampered by transmission errors. We will develop a suitable error-resistant stereoscopic video transmission system using the results from this investigation.

### 3.4. Subjective Method and Testing Conditions

A subjective assessment is the most efficient way to assess the apparent quality of a video that has been received but has had network faults that have been covered up distort it. The 32-inch Samsung 3D TV utilized for the subjective evaluation came with an ACTIVE shutter glass from Nvidia 3D vision that was necessary to see 3D content. In accordance with ITU-R BT.500.13 [38] and the VQEG HDTV [39] test plan, the ideal viewing distance was set to be three times the display’s height, which was one meter from the wall. The room lighting was also adjusted so that background light was limited to no more than 15% of the display’s illumination. The sole ambient illumination came from the wall behind the monitor, and the light source was 6500 $K^{0}$. The monitor was placed behind a wall so that the light coming from it did not shine directly on the audience and did not exceed 5% of the display’s maximum brightness (when used as a stereo monitor).

According to ITU-R BT.2021 [40], stereoscopic films may be evaluated subjectively using a number of human perceptual criteria, including video quality and visual comfort. The evaluation sessions were carried out using the Double Stimulus Continuous Quality Scale (DSCQS) methodology. In each trial, distorted and undistorted movies were shown to the audience in a random sequence. Participants were then asked to rate the videos’ overall video quality and comfort on a range of continuous measures from 1 to 5. With regard to the other rating criteria, scores that were close to 0 denoted the lowest quality or the most miserable situation, while scores of 5 denoted excellent video quality or a very comfortable position with regard to visual comfort. The approach used for the subjective investigations received clearance from the University of New South Wales’ Human Research Advisory Panel (approval number: A-13-41) for use in human research.

### 3.5. Experimental Datasets and Training Sessions

**Figure 9:**
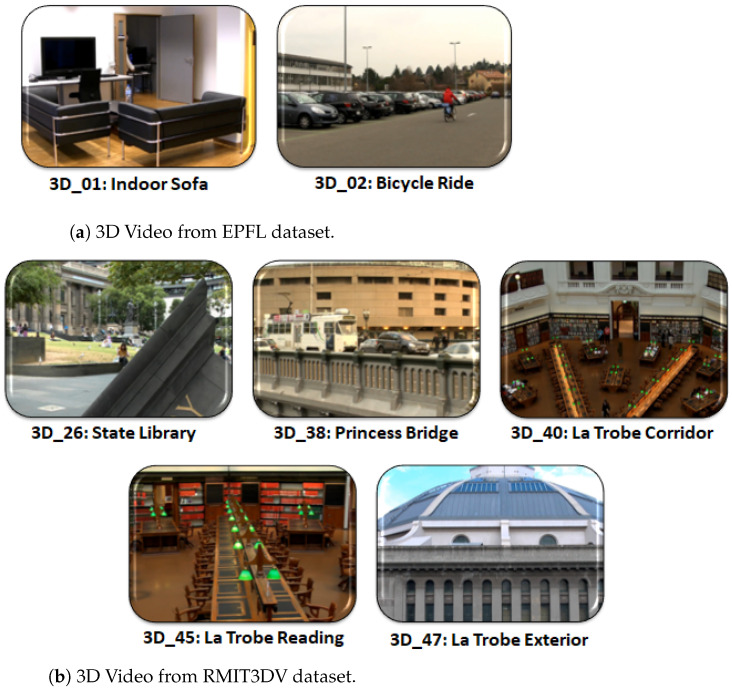
Video datasets: (**a**) EPFL (École Polytechnique Fédérale de Lausanne)-provided 3D video dataset; (**b**) RMIT3DV (Royal Melbourne Institute of Technology)-provided 3D video dataset.

The RMIT3DV [41] and EPFL [42] video datasets and seven distinct stereoscopic sequences (3D_01, 3D_02, 3D_26, 3D_38, 3D_40, 3D_45, and 3D_47) were employed in our experiment (Figure 9). All had various graphical components, such as camera and object motions and textures and had a 10-second playing length, full HD resolution of 1920×1080, and 25 frames per second. The first two sequences, 3D_01 and 3D_02, from the EPFL dataset, as shown in Figure 9a, depicted individuals lounging on couches inside rooms and riding bicycles on roads, respectively. The remaining images, 3D_26, 3D_38, 3D_40, 3D_45, and 3D_47 from the RMIT3DV collection, as in Figure 9b, were referred to as State Library, Princes Bridge, La Trobe Corridor, La Trobe Reading, and La Trobe Exterior, respectively. These films included diverse low-to-high 3D depth perceptions as well as distinct movements.

**Figure 10:**
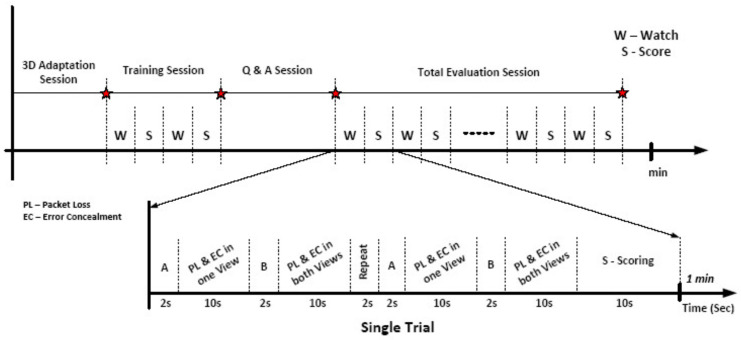
Timelines of evaluation session and the single trial.

As illustrated in Figure 10, a training session was held before the final test to show viewers how the subjective testing method, which had been approved by the UNSW Ethics Panel, worked. It was explained that, because they were expected to give scores based on videos that were simulated using the benchmark JM reference system, they might have some minor problems while watching them, such as binocular rivalry phenomena and visual discomfort, which could cause eye strain, headaches, nausea, and/or visual fatigue as well as affecting the overall quality of the video. After hearing about the binocular rivalry artifact and how it affects depth perception, the people who watched the video gave their written permission and took part in the subjective tests. After this briefing, there was 1-minute training on watching videos (W) and scoring (S). After that, there was a time for questions and answers to clear up any confusion about how the scores were made. Last but not least, the evaluation period was conducted and finished according to the Double Stimulus Continuous Quality Scale (DSCQS) standard. As shown in Figure 10, each one-minute trial was composed of one W and S where a 10-second video with packet loss and error concealment in one view or both views were played one after another, which were named A or B. The process was repeated again and was ended by scoring the videos.

### 3.6. Objective Video Quality Assessment

Packet loss and its effect on video signal quality may be evaluated by objective testing. This data can be used to optimize network settings, enhance video encoding parameters, or make other adjustments to provide the highest-quality video signal. To validate the packet loss simulation in our experiment, we employed objective video quality measurements. To accurately show the measure, both full-reference and no-reference quality evaluation criteria have been utilized. The peak signal-to-noise ratio (PSNR) [43], mean square error (MSE) [43], and Multiscale Structural Similarity Index Measure (MS-SSIM) [44] were used as full-reference criteria, while the Naturalness Image Quality Evaluator (NIQE) [45] was used to blindly estimate the quality without the source video (no-reference). These two distinct methodologies will assure the applicability of subjective quality assessment for simulated and transmitted 3D videos.

The Mean Squared Error (MSE) [43] is a measure of how well a regression model fits the data. It measures the average of the squared differences between the predicted values and the actual values. The main advantage of using MSE is that it gives more weight to larger errors, which is useful in situations where larger errors are more significant than smaller ones. However, it can also be sensitive to outliers in the data, as they can significantly increase the value of the MSE. The metric can be computed by using the following formula:

(4) $$d\left(X,Y\right)=\frac{\sum_{i=1,j=1}^{m,n}{\left(Y_{i,j}-X_{i,j}\right)}^{2}}{mn}$$

where *m*—video width, *n*—video height, and image data are in range [0, 1].

The Peaksignal-to-Noise Ratio (PSNR) [43] metric depends only on the difference of original and distorted, and more precisely, only on the L2-norm of this difference. The total PSNR is an aggregated value that considers all processed frames as a single huge image and then calculates the PSNR. Unlike the MSE, the metric has logarithmic scale and can be calculated using the following formula:

(5) $$PSNR=10\cdot log_{10}\frac{MaxErr^{2}\cdot w\cdot h}{\sum_{i=1,j=1}^{w,h}\left(x_{i,j}-y_{i,j}\right)}$$

where MaxErr—maximum possible absolute value of color component (MaxErr=1 in VQMT), *w*—video width, and *h*—video height.

The Multiscale Structural Similarity Index Measure (MS-SSIM) [44] is a method for measuring the structural similarity between two images. The MS-SSIM method operates by breaking down the images into multiple scales and comparing the structural similarity at each scale. This is performed by first applying a series of Gaussian filters to the images, each with a different standard deviation, to create a set of smoothed images at different scales. Then, the SSIM index is computed for each pair of corresponding images at each scale, and the results are combined into a single overall score.

The Naturalness Image Quality Evaluator (NIQE) [45] is a no-reference quality assessment method, meaning it does not require a reference image to evaluate the quality of an image. Instead, it uses statistical models to analyze the naturalness of the image by measuring the deviation of the image statistics from natural image statistics. The NIQE algorithm extracts several features from an image, including statistical measures such as mean, variance, and skewness, and applies a set of regression models to these features to obtain a quality score. The score ranges from 0 to 100, where higher scores indicate better image quality. NIQE has been found to be effective in assessing the quality of images and has been used in various applications, such as image compression, restoration, and enhancement.

## 4. Results and Discussions

A total of 22 viewers who were not familiar with the ITU-R BT.2021 standard were asked to subjectively evaluate the overall video quality and the sensation of visual comfort associated with several pairs of video materials. The criteria for visual discomfort included feelings of nausea, eye strain, double vision, and headaches. The viewers were asked to rate the videos on a scale that ranged from one to five points. To construct exact MB losses in both views, we used the same parameters, the CRF, fixed length slice, motion vector search method, etc., to encode both the left and right views of a stereoscopic video and then applied the same loss rate to both video streams. Since the videos were encoded in the NAL packet format, the exact numbers of NAL unit sequences and equal quantity of NAL units were discarded from both video streams using the H.264/AVC video stream analyzer [36,46], an approach that enabled the same MB error to be maintained in both views. After the simulated network loss, the videos were decoded and the same error concealment approach was applied to both distorted views. However, as it was a major challenge to maintain equal MB losses and exact error propagation in both views, with the losses sometimes deviated and misplaced due to disparity, the accuracy of the final experimental results was affected.

In each trial, error concealment in one and two views were represented as A and B, respectively. The two videos were repeated and then, during a 10 s period, the viewers rated each one. Therefore, the total duration for each trial was 60 s, with the timing of each experiment shown in Figure 10. Packet losses of 1% and 3% were used and error concealment was performed for each video dataset. In the case of error concealment in both views, the order of the playback of the packet losses and error concealment in one and two views varied in different trials. To refresh the error bit stream and re-synchronize the decoder after an error, different group of picture (GOP) sizes (intervals between I-frames) were used in the encoded videos, which led to viewers observing artifacts with both short and long durations. For our experiment, for a short duration, we specified the occurrence of an I-frame every 25 frames (1 s), which meant that, for short-duration sequences, the decoder could refresh its bit stream every second and, for long-duration ones, every 125 frames (i.e., every 5 s). As for every video sequence, there were 4 experiments with short or long durations and 1% or 3% packet losses; so, there was a total of 28 trials to be scored. The motivation for using these different packet loss rates and artifact durations was to determine viewers’ different perceptions based on their subjective scores in terms of video quality and visual comfort for these 3D videos.

### 4.1. Mean Opinion Score and Confidence Interval

The scores given by observers are averaged to produce the mean opinion score (MOS) and confidence intervals (CI) as indicated below:

(6) $$\begin{array}{c} {\hat{U}}_{jkr}=\frac{1}{N}\sum_{i=1}^{N}U_{ijkr} \end{array}$$

Here, ${\hat{U}}_{jkr}$ is the score of observer *i* for the degradation *j* in video sequence *k* and repetition *r*, and *N* is the number of observers. To better assess the accuracy of the results, as it is desirable to combine a CI with each MOS, we used a 95% one in

(7) $$\begin{array}{c} {CI}_{mean}=\left\{\begin{array}{c} {CI}_{lower}={\hat{U}}_{jkr}-\delta_{jkr}\\ {CI}_{upper}={\hat{U}}_{jkr}+\delta_{jkr} \end{array}\right. \end{array}$$

(8) $$\begin{array}{c} \delta_{jkr}=\left(N_{t-val}\times {SE}_{mean}\right) \end{array}$$

where $\delta_{jkr}$ is the standards deviation of mean ${\hat{U}}_{jkr}$, depending on the critical t-value ($N_{t-val}$) and standard error of the mean ${SE}_{mean}$. For sample size N=22, the degree of freedom is df=N−1=21 with $N_{t-val}=1.721$ according to the one-tailed test t-table for a 95% CI.

In the final subjective assessment session, viewers were asked to record their observations of video quality and visual comfort in each trial. After considering all the scores between 0 and 5, the MOSs were calculated by Equation (6), with those for video quality and visual comfort for 1% and 3% packet losses (Pkt Loss) shown in Table 1 and Table 2, respectively. The table shows the MOSs for the 7 stereoscopic video datasets used to study the impact of MB-loss error concealment. The perceptual characteristics for both video quality and visual comfort were measured for short and long GOP video sequences. In both Table 1 and Table 2, it can be seen that both views achieved higher MOSs than single ones because, in the latter, binocular rivalry artifacts were induced in 3D perception.

**Figure 11:**
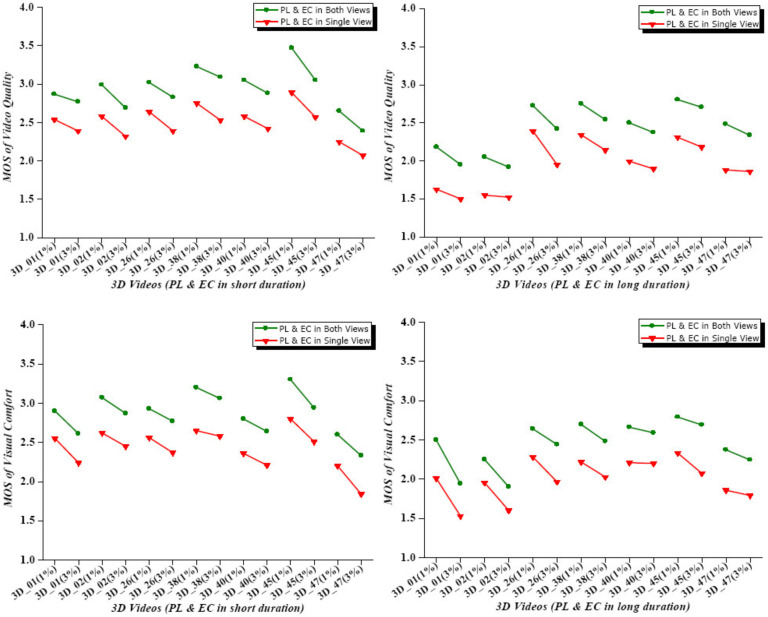
Video quality and visual comfort charts: lines indicate MOS variations for different packet losses and plot different artifact durations.

A comparison of the scores is shown in the graph in Figure 11 in which the *x*-axis indicates the 1% and 3% packet losses (PLs) and error concealments (ECs) for the 7 different stereoscopic videos and the *y*-axis the viewer’s MOSs from 1 to 5 (a maximum MOS of 4.0 is shown as none of the experiments exceeded this value). Different loss rates for the same video are connected by a solid line, with the green and red ones indicating the connections between the PL and EC values for both views and a single one, respectively. The significant differences between the green and red lines show that there was a clear division between the viewer’s scores obtained from the approaches for single and both views. Moreover, the successive red lines demonstrate that binocular rivalry in one view generated much greater visual degradation for viewers than that in both views (green lines).

**Figure 12:**
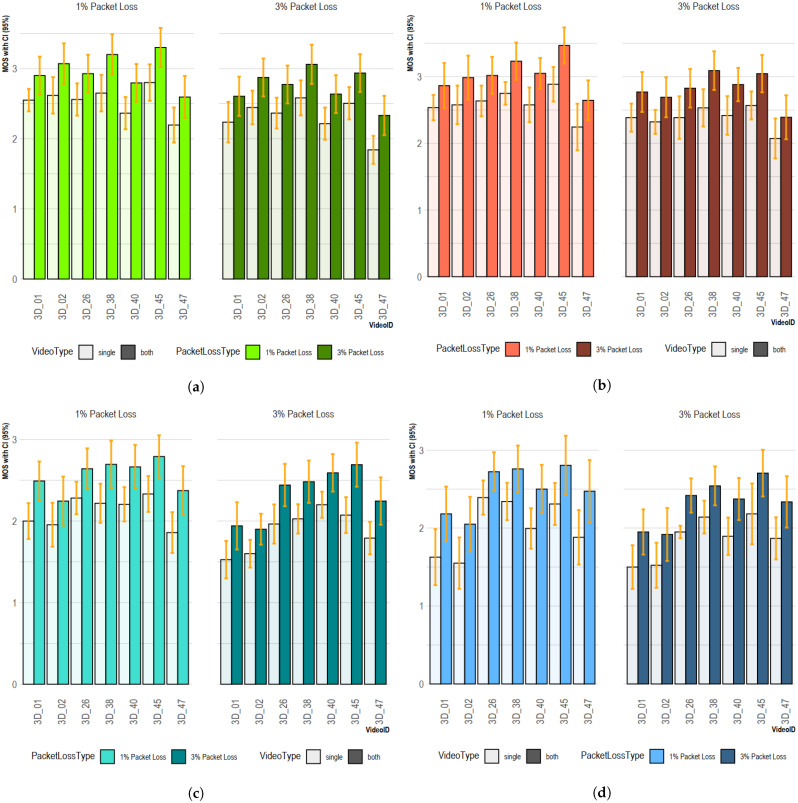
The 95% CIs for MOSs of visual comfort and video quality for short- and long-duration (GOP) videos with packet loss and error concealment. (**a**) Visual comfort: short-duration videos. (**b**) Video quality: short-duration videos. (**c**) Visual comfort: long-duration videos. (**d**) Video quality: long-duration videos.

In order to analyze the accuracy and confidence of the given scores, 95% CIs were calculated using Equation (8). In general, a small range of CIs indicates that, if the experiment was repeated, we could be confident of obtaining a similar result whereas a large one suggests less certainty and implies that results need to be collected from more people as CIs are influenced by the number of people participating in the experiment. Using Equation (8), the 95% CI was calculated from the MOSs of 22 observers and, in most cases, lay between ±0.3, which indicated high confidence in the viewers’ scores. Most evident was the viewers’ CIs for single-view error concealment which, according to our experiment, were between ±0.23 for most cases and less than those for error concealment in both views. This also indicated viewer certainty in terms of the binocular rivalry artifacts imposed by single-view error concealment, as shown in Figure 12, where 1% and 3% PLs and ECs of the 3D_01 dataset are shown as 01_1B and 01_3B for both views and 01_1S and 01_3S for a single one, respectively. The same naming convention is maintained for the other experimental video datasets.

### 4.2. Objective Quality Assessment

Video quality can be significantly impacted by network packet loss, which can lead to visual discomfort, artifacts, and other issues. We used an MSE-, PSNR-, MS-SSIM-, and NIQE-based technique to estimate the objective testing for network packet loss in videos. The method allows us to determine if the simulation environment produced the necessary losses in the left and right views of a stereoscopic video.

**Figure 13:**
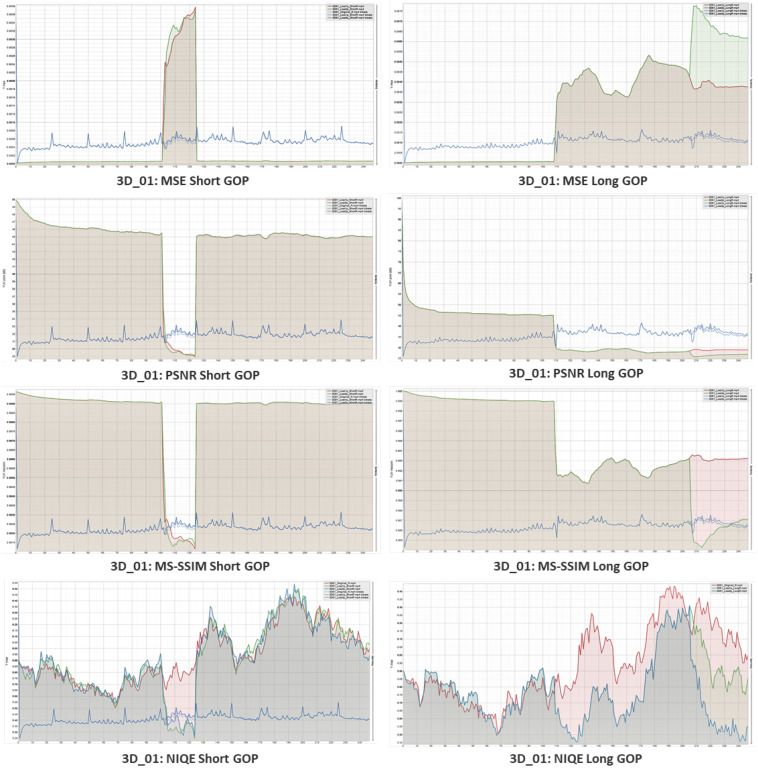
Objective quality assessment of simulated 3D_01 video sequence. Left column shows the metrics for short GOP and right column shows the metrics for long GOP. *X*-axis shows the frame numbers and *Y*-axis shows the metric for individual quality measure.

**Figure 14:**
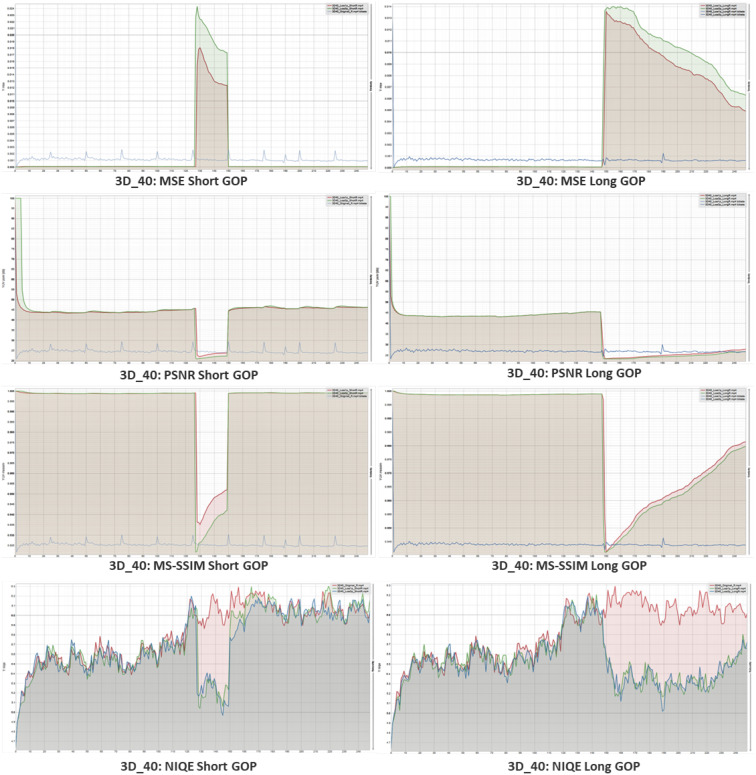
Objective quality assessment of simulated 3D_40 video sequence. *X*-axis shows the frame numbers and *Y*-axis shows the metric for individual measure.

Both the short GOP videos and the long GOP videos underwent a comparison analysis, as seen in Figure 13 and Figure 14. For instance, in the following images, we have two stereo films, 3D_01 and 3D_40. The MSE, PSNR, and MS-SSIM were used as full-reference metrics for objective assessment. The red line in the graph represents a 1% packet loss, whereas the green line represents a 3% packet loss. In a sense, the three percent error ended up being greater than the one percent loss. Moreover, the blue line depicts the bit rate of the videos, where I-frames contain more bits, which can be seen as a continuous high pulse in between the video frame bit rates in the short GOP video.

During 3 percent packet loss, the MSE experiences a greater loss than when 1 percent of packets are lost. Meanwhile, the PSNR declines when packet losses occur and significantly worsens for extended GOP when there is no I-frame present during encoding. The same outcomes in terms of structural difference brought on by mistake are likewise shown by the MS-SSIM. The score that confirms the video has acceptable losses for greater inspection during the subjective assessment is obviously reduced by the higher proportion of packet losses. The no-reference video quality metric NIQE was then used to measure a video’s naturalness. The red line in this example displays the original encoded video without any degradation. In contrast, the green line indicates the score for 1% pkt loss and the blue line indicates the score for 3% pkt loss.

If there is any ambiguity or mismatch in the bit rate or an unwelcome drop or rise in the video, the video is rejected and re-run through the simulation environment in order to pass the objective assessment part. To determine if loss rates are correctly caused, the identical approach is applied to all experimental video datasets. With the help of all of these assessments, we would be able to verify that the simulation-based methodology is maintained throughout the whole study.

### 4.3. Analysis of Student’s *T*-Test and Statistical Significance

In our approach, we used the paired-sample *t*-test to compare the means of two variables for a single group by computing the differences between the values of these variables for each case and testing whether the average differed from zero. This sample was then used to compare the mean of a single sample of scores with a known or hypothetical population mean $\left(H_{mean}\right)$ [47]. Based on the null hypothesis, we computed whether one group of samples was better than the other through a t-probability distribution, where *N* was the equal number of samples in each distribution and $S_{1},S_{2}$ the two sample groups. The $T_{test}$ was the Student-*t* test value used to express the CIs for a set of data and compare the results obtained from different experiments. The aim was to state the possible range of true mean, ${\hat{U}}_{true}$, from the measured mean ${\hat{U}}_{jkr}$ with probability.

(9) $$\begin{array}{c} {\hat{U}}_{true}={\hat{U}}_{jkr}\pm T_{test}\frac{S_{jkr}}{\sqrt{N}} \end{array}$$

(10) $$\begin{array}{c} T_{test}=\frac{X_{mean}-H_{mean}}{\sqrt{\frac{\sum_{i=1}^{N}{\left(X_{i}-X_{mean}\right)}^{2}}{N(N-1)}}} \end{array}$$

where $X_{i}$ represents the differences between sample groups $S_{1}$ and $S_{2}$, and $X_{mean}$ represents the mean of these differences $\left(X_{mean}=\frac{X_{i}}{N}=\frac{\sum_{i=1}^{N}\left(S_{1i}-S_{2i}\right)}{N}\right)$. The calculated Student’s *t*-test value $T_{test}$ was compared with the critical (threshold) value $T_{th}$ corresponding to the degree of freedom (df) calculated as df=N−1 and then the confidence level, i.e., 90% or 95%, was chosen. If $T_{test}\geq T_{th}$, $H_{0}$ was rejected; otherwise, $H_{0}$ was accepted. The critical threshold $T_{th}$ was estimated from the probability of obtaining test statistics called the *p*-value. The obtained $T_{test}$ value, was used to calculate the *p*-value to determine how confident we are that the difference between the two distributions was significant. In statistics, the least degree of significance that may be observed while still rejecting the null hypothesis is referred to as the *p*-value. To put it another way, the less significant it was, the stronger the evidence was that supported the alternative theory. The value of the integral has to be found by the following integral in order to satisfy the area under the t-probability distribution in the interval (a,b). In the case when the degree of freedom, df=21, and Gamma, the gamma function defined by the integral [47] was used,

(11) $$\begin{array}{c} P-Value=\int_{a}^{b}f\left(T_{test}\right)dT_{test} \end{array}$$

The validity of a claim made about a population may be investigated via the use of hypothesis testing. This experiment compared the error concealment in both views ($S_{1}$) to the error concealment in just one view ($S_{2}$). We performed an analysis on the *t*-test scores that were derived from Equation (11) and estimated the *p*-value using Equation (10). Considering that the suggested method was inferior to those already in use for concealing, the new population mean $\left(X_{mean}\right)$ ought to have been lower than or equal to the hypothetical population mean $\left(H_{mean}\right)$, as shown here:

$$\mathrm{Null}\mathrm{Hypothesis}\;H_{0}:X_{mean}\leq H_{mean}$$

$$\mathrm{Alternative}\mathrm{Hypothesis}\;H_{A}:X_{mean}>H_{mean}$$

The findings that supported the alternative hypothesis revealed that the proposed strategy was superior to other ways that are already in use. We were only interested in one side of the distribution; thus, in order to compute the result of the *t*-test, we used what is known as a one-sided test, also called a one-tailed test. Although a lower *p*-value (usually ≤0.05) gave strong evidence against the null hypothesis, as this hypothesis could be rejected, there would be a statistically significant difference between the two populations, which would aid in the comprehension and demonstration of this difference. Table 3 classifies statistical significance into numerous categories based on different *p*-values.

The MOSs obtained from 22 participants were then compared using a one-tailed paired (dependent) *t*-test, and the *p*-values specified in Equation (11) were computed to establish the statistical significance between the 2 populations, taking into account the null hypothesis that MB error concealment in both views is worse than that in a single view. Accepting the null hypothesis requires a *p*-value ≤0.05, degrees of freedom (df) of (N−1)=21, and $T_{test}$ ≤1.721 for a 95% confidence interval. Otherwise, it would be rejected and replaced with a substitute. On the basis of the MOSs, the *t*-test and *p*-value scores were generated to examine the statistical significant values of error concealments in single and both views for short and long GOP sequences, with the results shown in Table 4 and Table 5 demonstrating that there were varying degrees of significance for different *p*-values, as indicated in Table 3.

Table 4 and Table 5 provide the results of the statistical significance analysis of error concealments in both views against one view, together with their estimated *t*-test and *p*-value scores and distinct significant criteria assigned depending on the threshold levels established in Table 3. We may examine the psychovisual changes in the MOSs induced by binocular competition in 3D depth perception based on the statistical significance thresholds. For short GOP sequences, 28 comparison studies were undertaken, and statistical significance was attained for both 1% and 3% packet losses in all video datasets. Ten of them displayed great statistical significance by rejecting the null hypothesis unequivocally. In addition, the statistical significance values of all the video datasets, including 13 very significant ones, demonstrated that, for a short GOP sequence, MB error concealment is significantly superior in both perspectives compared to a single view in terms of visual comfort and video quality. Since 26 of the 28 experiments unambiguously rejected the null hypothesis for lengthy GOP sequences using VSS or ExSS, the effect of binocular rivalry on single-view error concealment was proven.

Moreover, more ExSS in the 3% than 1% packet losses explained that binocular rivalry artifacts affected them more due to more artifacts being induced for packet losses and error concealment afterwards. Even in the matter of comparing Table 4 and Table 5, it can be found that more VSS and ExSS are analyzed for longer GOP than short GOP. It also explains that if artifacts such as binocular rivalry sustain a longer period of time it creates more visual discomfort in 3D QoE.

It was found that MB-loss error concealment in both views rejected the null hypothesis more often and supported the alternative hypothesis that error concealment in both views is superior to that in one. The null hypothesis stated that error concealment in one view is superior to that in the other two views. However, it was found that this was not the case. Instead, it supported the alternative hypothesis that error concealment in both views is superior to that in one. The $T_{test}$ results offered quantitative evidence for rejecting the null hypothesis, in contrast to the statistical evidence that was given by the MOSs and the Student’s *t*-test analysis. As a result of this, a severe binocular rivalry artifact can be seen in the stereoscopic video’s MB-loss error concealment in a single view. This is the leading cause of viewers’ lack of visual comfort when watching transmitted or broadcasted stereoscopic videos, which were subjected to real-life simulated transmitting conditions and observed in a standardized assessing environment. Stereoscopic videos were tested using real-life simulated transmitting conditions and were evaluated in a prescribed scientific environment.

## 5. Conclusions

This study identified the binocular sensitivity and rivalry effects of error concealment and showed that 2D EC methods are not acceptable for 3D videos without adjustments. The binocular rivalry artifact’s effect on stereoscopic video transmission was tested using independently encoded streams. For a single view, the MOS score for packet loss and error concealment falls between 2.25 and 2.75, while on both views the range is 2.65–3.37 for a 1% point loss in the short GOP sequences. The same holds for a 3% packet loss, such as 2.39 to 3.09 for both and 2.07 to 2.53 for single-view artifacts. Due to the adverse effect of binocular rivalry, a single view may score lower than both views. Additional analyses of the visual comfort of long GOP or MOS suggest similar concerns. To make it profound, we expanded the method to statistical t-test analysis and discovered that the negative effect is so pervasive that, in the vast majority of cases, the comparisons between single and both perspectives are statistically significant (SS) to extremely significant (ExSS): 13 out of 14 (93%) video quality metrics for short GOP are VSS and ExSS; 10 out of 14 (71%) visual comfort measurements are VSS and ExSS. In addition, the score for lengthy GOP increases to 100% for video quality and 86% for visual comfort. However, utilizing this basic approach, binocular rivalry disrupts the HVS and produces eye strain, headaches, nausea, weariness, etc. This work analyzed binocular rivalry to create a robust error-resilient 3D video communication system without perceptual ambiguity or rivalry. This investigation will enable researchers to develop real-time error concealment algorithms for 3D videos that do not degrade visual perceptual comfort.

## Figures and Tables

**Table 1:** MOS of video quality for macroblock-loss error concealment.

MOS of Video Quality								
Experimental Videos	Short GOP				Long GOP			
Datasets	1% Packet Loss		3% Packet Loss		1% Packet Loss		3% Packet Loss	
	Both	Single	Both	Single	Both	Single	Both	Single
3D_01	2.87	2.54	2.77	2.39	2.18	1.63	1.95	1.50
3D_02	2.99	2.58	2.69	2.32	2.05	1.55	1.92	1.52
3D_26	3.02	2.64	2.83	2.39	2.72	2.39	2.42	1.95
3D_38	3.23	2.75	3.09	2.53	2.76	2.34	2.54	2.14
3D_40	3.05	2.58	2.88	2.42	2.50	1.99	2.37	1.89
3D_45	3.47	2.89	3.05	2.57	2.81	2.31	2.71	2.18
3D_47	2.65	2.25	2.39	2.07	2.47	1.88	2.34	1.87

**Table 2:** MOS of visual comfort for macroblock-loss error concealment.

MOS of Visual Comfort								
Experimental Videos	Short GOP				Long GOP			
Datasets	1% Packet Loss		3% Packet Loss		1% Packet Loss		3% Packet Loss	
	Both	Single	Both	Single	Both	Single	Both	Single
3D_01	2.91	2.55	2.61	2.24	2.49	2.00	1.94	1.53
3D_02	3.07	2.62	2.87	2.45	2.25	1.96	1.91	1.60
3D_26	2.93	2.56	2.77	2.37	2.64	2.28	2.44	1.96
3D_38	3.20	2.65	3.06	2.58	2.70	2.22	2.48	2.03
3D_40	2.79	2.36	2.64	2.21	2.66	2.21	2.59	2.20
3D_45	3.32	2.80	2.94	2.51	2.79	2.33	2.69	2.07
3D_47	2.59	2.20	2.33	1.84	2.37	1.86	2.25	1.79

**Table 3:** Categories of statistical significance based on *p*-value.

Type	Significance	*p*-value	Comment
ExSS	Extreme statistical significance	**≤0.001**	Extremely strong evidence against the null hypothesis
VSS	Very statistical significance	**≤0.01**	Very strong evidence against the null hypothesis
SS	Statistical significance	**≤0.05**	Strong evidence against the null hypothesis
NqSS	Not quite SS	**≤0.1**	Marginal
NSS	Not SS	**>0.1**	Fail to reject null hypothesis

**Table 4:** Statistical significance analysis of video quality and visual comfort of error concealments in short GOP sequences.

Comp. of Single and Both Views		Video Quality			Visual Comfort		
**PLR (%)**	**Video Datasets**	** $T_{\mathrm{test}}$ **	***p*** **-Value**	**Stat. Sig.**	** $T_{\mathrm{test}}$ **	***p*** **-Value**	**Stat. Sig.**
	Indoor Sofa	2.5903	0.00854	VSS	2.1841	0.02022	SS
	Bicycle Ride	3.6643	0.00072	ExSS	3.4265	0.00127	VSS
	State Library	2.8603	0.00469	VSS	2.6045	0.00828	VSS
1% PL and EC	Princess Bridge	4.4514	0.00011	ExSS	3.1701	0.00231	VSS
	La Trobe Corridor	4.0153	0.00031	ExSS	2.3050	0.01574	SS
	La Trobe Reading	3.0548	0.00351	VSS	3.4056	0.00133	VSS
	La Trobe Exterior	3.3775	0.00142	VSS	2.4221	0.01228	SS
	Indoor Sofa	3.8847	0.00043	ExSS	3.7059	0.00065	ExSS
	Bicycle Ride	2.6516	0.00746	VSS	2.5249	0.00984	VSS
	State Library	2.6291	0.00784	VSS	2.7456	0.00606	VSS
3% PL and EC	Princess Bridge	6.1220	<0.00001	ExSS	3.9772	0.00034	ExSS
	La Trobe Corridor	2.8565	0.00047	ExSS	3.7742	0.00056	ExSS
	La Trobe Reading	3.7413	0.00060	ExSS	3.1259	0.00255	VSS
	La Trobe Exterior	1.8838	0.03676	SS	2.1597	0.02125	SS

PLR = packet loss rate, PL = packet loss, EC = error concealment, Stat. Sig. = statistical significance.

**Table 5:** Statistical significance analysis of video quality and visual comfort of error concealments in long GOP sequences.

Comp. of Single and Both Views		Video Quality			Visual Comfort		
**PLR (%)**	**Video Datasets**	** $T_{\mathrm{test}}$ **	***p*** **-Value**	**Stat. Sig.**	** $T_{\mathrm{test}}$ **	***p*** **-Value**	**Stat. Sig.**
	Indoor Sofa	2.6845	0.00694	VSS	2.3228	0.01516	SS
	Bicycle Ride	2.5989	0.00828	VSS	3.0603	0.00297	VSS
	State Library	4.5819	0.00008	ExSS	4.4152	0.00012	ExSS
1% PL and EC	Princess Bridge	3.4693	0.00115	VSS	3.1775	0.00227	VSS
	La Trobe Corridor	5.0294	0.00003	ExSS	2.0932	0.02433	SS
	La Trobe Reading	3.1865	0.00222	VSS	5.6085	<0.00001	ExSS
	La Trobe Exterior	4.3416	0.00014	ExSS	3.1509	0.00241	VSS
	Indoor Sofa	2.5493	0.00934	VSS	2.8627	0.00466	VSS
	Bicycle Ride	3.3732	0.00144	VSS	2.9815	0.00356	VSS
	State Library	4.5425	0.00009	ExSS	2.6932	0.00681	VSS
3% PL and EC	Princess Bridge	2.7983	0.00054	ExSS	3.5398	0.00097	ExSS
	La Trobe Corridor	4.1138	0.00025	ExSS	4.2103	0.00019	ExSS
	La Trobe Reading	3.1755	0.00228	VSS	3.2616	0.00187	VSS
	La Trobe Exterior	3.8512	0.00046	ExSS	3.5696	0.00091	ExSS

PLR = packet loss rate, PL = packet loss, EC = error concealment, Stat. Sig. = statistical significance.

## Data Availability

The full experimental video sequences (both left and right views), the produced raw data after the subjective experiments, as well as experimental codes for simulating transmission error, error propagation, and analysis of the macroblock losses in the video frames can now be found at the following figshare repositories: https://doi.org/10.6084/m9.figshare.20985676 (accessed on 7 September 2022), https://doi.org/10.6084/m9.figshare.21027580 (accessed on 7 September 2022), https://doi.org/10.6084/m9.figshare.20986519 (accessed on 7 September 2022).

## Appendix A. Encoding and Decoding Process

In our tests, the command initiated the encoding process below:

#lencod.exe−d<configurationfile>

where the configuration file included all the settings for parameters required to encode the video sequence and create the bit stream. Below is a brief summary of the specific parameter values we utilized in our experiment to encode the videos: Table A1. In our experimental movies, other elements, including the constant rate (CRF), were employed to keep the video quality consistent. Just the most recent encoded frame was utilized as the reference frame, and the search range for motion estimation was set to ±32 pixels. Subpixel precision and sub-MB motion compensation were also permitted. The loss was induced by applying the following command:

#rtp_loss.exe<input_file><output_file><loss_ratein%>[skippackets]

**Table A1:** Parameters of encoder configuration file.

InputFile	= “input.yuv”	# Input sequence
FramesToBeEncoded	= 250	# No of frames to be coded
FrameRate	= 25.0	# Frame rate (1–100)
OutputFile	= “output.264”	# Bitstream
ProfileIDC	= 100	# Profile IDC
IntraPeriod	= 25	# Period of I-picture
IDRPeriod	= 0	# Period of IDR picture
QPISlice	= 28	# Quant.param for I slices
QPPSlice	= 28	# Quant.param for P slices
SearchRange	= 32	# Max search range
NumberReferFrm	= 1	# Previous frames for inter-motion search (0–16)
Log2MaxFNumM4	= −1	# −1
SymbolMode	= 1	# Entropy coding
OutFileMode	= 1	# Output file mode, 1:RTP
SliceMode	= 1	# Slice mode (1=fxd mb)
SliceArgument	= user-defined	# Slice argument
num_slice_grp_m1	= 0	# No. of slice groups - 1
slice_grp_map_typ	= 0 to 6	# Different group types
ConfigFileName	= “sg0conf.cfg”	# slice_group_map_type 0,2,6

Finally, the decoding of the error-impaired videos was initiated by the following command:

#ldecod.exe<configurationfile>

where the user’s experimental needs may be taken into account while setting up some of the decoding settings, which were specified in the configuration file. These criteria include some of the following. Table A2 lists specific decoder configuration file parameters.

**Table A2:** Parameters of decoder configuration file.

InputFile	= “test.264”	# H.264/AVC coded bitstream
OutputFile	= “test_dec.yuv”	# Output file, YUV/RGB
RefFile	= “test_rec.yuv”	# Ref sequence (for SNR)
WriteUV	= 1	# Write 4:2:0 chroma components
FileFormat	= 1	# NAL(0=AnnexB,1=RTP packets)
ConcealMode	= 1	# Err concealment, 0: Off, # 1: FrameCopy, 2: MotionCopy
